# Metagenomic data of fungal community in Kongsfjorden, Arctic using Illumina next generation sequencing

**DOI:** 10.1016/j.dib.2018.12.026

**Published:** 2018-12-13

**Authors:** Farha Arakkaveettil Kabeer, T. Jabir, K.P. Krishnan, Mohamed Hatha Abdulla

**Affiliations:** a Department of Marine Biology, Microbiology & Biochemistry, School of Marine Sciences, Cochin University of Science and Technology (CUSAT), Kochi, India; b National Centre for Polar and Ocean Research, Headland Sada, Vasco-da-Gama, Goa, India

## Abstract

The data represents the diversity and distribution of fungal communities in Kongsfjorden, Arctic. The metagenomic DNA analysis was performed using next generation sequencing technology (Illumina MiSeq). Sequence data from amplified internal transcribed spacers (ITS) 2 region with fungal-specific primers exposed 83,417 sequences belonging to 7 operational taxonomic units (OTUs). Five of these OTUs belonged to Ascomycota, and one each to Basidiomycota and unclassified group. *Aspergillus, Candida, Emericella* and *Nakaseomyces* were the different genera identified and they belonged to the fungal orders *Helotiales, Eurotiales* and *Saccharomycetales.* The data explored the presence of important fungal communities in the Arctic marine ecosystem. Metagenome data is now available at NCBI under the Sequence Read Archive (SRA) database with accession no. SRP152688.

## Specifications table

**Table:** 

Subject area	*Biology*
More specific subject area	*Metagenomics*
Type of data	*Figure*
How data was acquired	*NGS sequencing on Illumina MiSeq platform*
Data format	*Raw data*
Experimental factors	*Marine sediment samples from Kongsfjorden, Arctic*
Experimental features	*eDNA isolation and sequencing of marine sediment samples from Arctic*
Data source location	*Two stations (78°55׳365N, 12°05׳642׳ E) (78°57׳454N, 11°49׳088 E) from Kongsfjorden, Arctic.*
Data accessibility	*Data is with this article.*
	*The data of this metagenome is available in the NCBI BioSample Submission Portal as Bioproject* PRJNA480134 *and SRA accession no.*SRP152688.
	https://www.ncbi.nlm.nih.gov/sra/?term=SRP152688
Related research article	*None*

## Value of the data

• The data provides valuable information of the diversity of fungal communities in the marine sediment of *Kongsfjorden*, Arctic.
• This metagenome data will be useful for the study and comparison of fungal communities across the Arctic.
• Profiling of fungal communities using Illumina technology provides a cost effective and an efficient in depth sequencing.
• The fungal metagenomic data exposes Arctic ecosystem as a potential source for bioprospecting novel extremozymes with industrial application.

## 1 Direct link to deposited data

Deposited data can be found here: https://www.ncbi.nlm.nih.gov/sra/?term=SRP152688.

## 2 Data

The data article explores the diversity of fungal communities in the sediments from Kongsfjorden, Arctic by using high throughput sequencing.

**Fig. 1:**
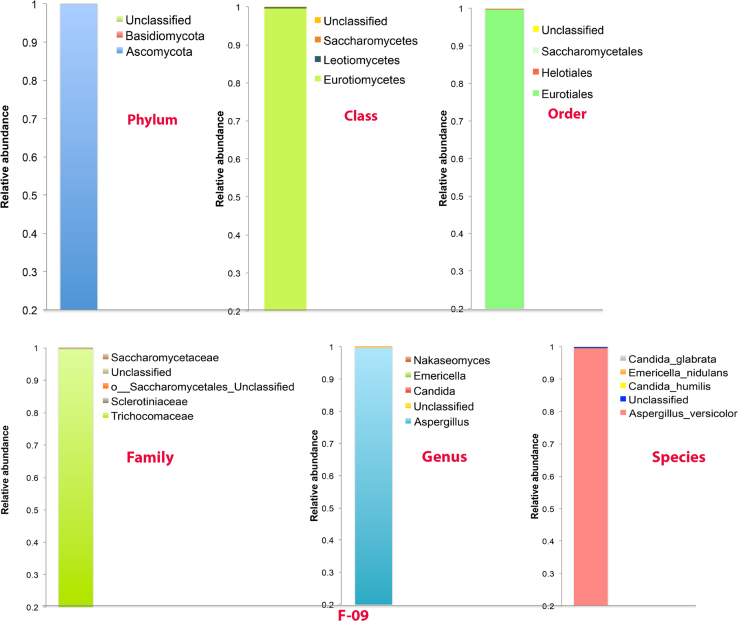
Relative abundance of fungi in the sample at different taxonomic levels (Phylum, Class, Order, Family, Genus and Species).

The preliminary sequencing of raw data has revealed 166,834 sequences with an average base pair length of 250. After quality optimization of data, 80,644 sequences were filtered from raw data. A total of seven fungal OTUs was identified and classified. These seven OTUs spanned 3 phyla, 4 classes, 4 orders, 5 families, 5 genera and 5 species. Ascomycota was the dominant phylum identified followed by *Basidiomycota* and unknown fungi. *Aspergillus* (99.57%) was the dominant genus among the 5 genera followed by unknown fungi (0.30%), *Candida* (0.10%), *Emericella* (0.03%) and *Nakaseomyces*. The most abundant species identified were *Aspergillus versicolor, Candida humilis, Emericella nidulans, Candida glabrata* and unknown spp. (Fig. 1). This is the first report on fungal diversity from the Arctic region using NGS technology based on Illumina sequencing approach.

## 3 Experimental design, materials and methods

Marine sediment samples collected from two different locations of Arctic Kongsfjorden – KG7 (78°55׳365 N, 12°05׳642׳ E) and KG5 (78°57׳454 N, 11°49׳088 E) as part of Indian Arctic Expedition 2017 were used for metagenomic DNA extraction (Power Soil^®^ DNA Isolation Kit (MO BIO Laboratories Inc., Carlsbad, CA, United States)). DNA from both samples was pooled together (F09) for sequencing. ITS-Amplicon sequencing was performed with Illumina MiSeq sequencing system (OmicsGen LifeSciences Pvt. Ltd.). Briefly, amplification of the ITS2 region was carried out using the forward and reverse primers with sequences "GTGAATCATCGARTC" and "TCCTCCGCTTATTGAT’’ respectively. After validation by Agilent 2100 Bioanalyzer (Agilent Technologies, Palo Alto, CA, USA), DNA libraries were quantified by Qubit 2.0 Fluorometer and loaded on Illumina MiSeq instrument (Illumina, San Diego, CA, USA). Sequencing was performed using a 2 × 300/250 paired-end (PE) configuration. MiSeq Control Software (MCS) embedded in the MiSeq instrument conducted image analysis and base calling. ITS rRNA data analysis was performed using QIIME data analysis package [1].
