# Supplementary material for: Metagenomic data of fungal community in Kongsfjorden, Arctic using Illumina next generation sequencing
Source: Data Brief. 2018 Dec 13;22:195–8. doi: 10.1016/j.dib.2018.12.026 (PMC6301978; doi:10.1016/j.dib.2018.12.026)
Supplement: Supplementary file 1 — Supplementary material [file mmc1.pdf]

Conflicts of Interest Statement

Journal: Data in Brief

Manuscript Title: Metagenomic data of fungal community in Kongsfjorden, Arctic using Illumina next generation sequencing

The authors whose names are listed immediately below certify that they have NO affiliations with or involvement in any organization or entity with any financial interest (such as honoraria; educational grants; participation in speakers' bureaus; membership, employment, consultancies, stock ownership, or other equity interest; and expert testimony or patent-licensing arrangements), or non-financial interest (such as personal or professional relationships, affiliations, knowledge or beliefs) in the subject matter or materials discussed in this manuscript.

Author names:

Farha Arakkaveettil Kabeer [1]

Jabir T [1]

Krishnan K. P [2]

Mohamed Hatha Abdulla [1]

Affiliations: [1]Department of Marine Biology, Microbiology & Biochemistry,

School of Marine Sciences,

Cochin University of Science and Technology (CUSAT), Kochi, India.

[2]National Centre for Antarctic and Ocean Research, Headland Sada, Vasco-da-Gama, Goa. 403 804, India.

This statement is signed by all the authors to indicate agreement that the above information is true and correct

Author’s name	Author's signature	Date
Farha Arakkaveettil Kabeer	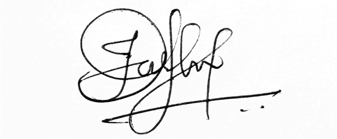	**27/10/18**
**Jabir T**	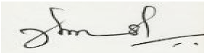	**27/10/18**
**Krishnan K. P**	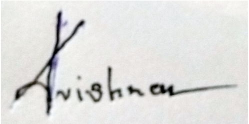	**28/10/2018**
**Mohamed Hatha Abdulla**	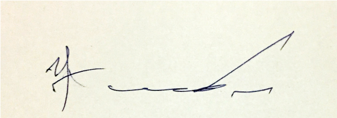	**27/10/18**
